# PCLO rs2522833-mediated gray matter volume reduction in patients with drug-naive, first-episode major depressive disorder

**DOI:** 10.1038/tp.2017.100

**Published:** 2017-05-30

**Authors:** R Igata, A Katsuki, S Kakeda, K Watanabe, N Igata, H Hori, Y Konishi, K Atake, Y Kawasaki, Y Korogi, R Yoshimura

**Affiliations:** 1 Department of Psychiatry, University of Occupational and Environmental Health, Kitakyushu, Japan; 2 Department of Radiology, University of Occupational and Environmental Health, Kitakyushu, Japan; 3 Department of Environmental Oncology, Institute of Ecological Sciences, University of Occupational and Environmental Health, Kitakyushu, Japan

## Abstract

Major depressive disorder (MDD) has been linked to differences in the volume of certain areas of the brain and to variants in the piccolo presynaptic cytomatrix protein (*PCLO*), but the relationship between *PCLO* and brain morphology has not been studied. A single-nucleotide polymorphism (SNP) in *PCLO*, rs2522833, is thought to affect protein stability and the activity of the hypothalamic–pituitary–adrenal axis. We investigated the relationship between cortical volume and this SNP in first-episode, drug-naive patients with MDD or healthy control subjects. Seventy-eight participants, including 30 patients with MDD and 48 healthy control subjects, were recruited via interview. *PCLO* rs2522833 genotyping and plasma cortisol assays were performed, and gray matter volume was estimated using structural magnetic resonance images. Among the individuals carrying the C-allele of *PCLO* rs2522833, the volume of the left temporal pole was significantly smaller in those with MDD than in healthy controls (family-wise error-corrected, *P*=0.003). No differences were detected in other brain regions. In addition, the C-carriers showed a larger volume reduction in the left temporal pole than those in the individuals with A/A genotype (*P*=0.0099). Plasma cortisol levels were significantly higher in MDD-affected C-carriers than in the healthy control C-carriers (12.76±6.10 vs 9.31±3.60 nm, *P*=0.045). We conclude that *PCLO* SNP rs2522833 is associated with a gray matter volume reduction in the left temporal pole in drug-naive, first-episode patients with MDD carrying the C-allele.

## Introduction

The pathophysiology of major depressive disorder (MDD) remains unclear. Monoamines, cytokines, neurotrophic factors and microcircuits are associated with MDD. Dysregulation of the hypothalamic–pituitary–adrenal (HPA) axis has also been described in patients with MDD. It has been reported that HPA axis-associated genes such as glucocorticoid receptor,^1^ corticotropin-releasing hormone (CRH) receptor type 1 (ref. 2) and FK506 binding protein 5 (ref. 3) are associated with MDD. The piccolo presynaptic cytomatrix protein (*PCLO*) gene is located on the chromosome 7q11-21. The protein product of the *PCLO* gene is localized at the cytoskeletal matrix of the presynaptic active zone, and plays an important role in monoaminergic neurotransmission in the brain.^4^ The single-nucleotide polymorphism (SNP) rs2522833, in which the major allele is an A and the minor allele is a C, substituting the usual hydrophilic, uncharged amino acid serine with charged alanine in the calcium-binding C2A domain of *PCLO*, may affect protein stability.^5^

A recent genome-wide association study revealed evidence for a role of C-allele (the risk allele) of SNP rs2522833 within the *PCLO* gene in the pathophysiology of MDD.^6^ In the Japanese population, the frequency of carriers of the rs2522833 C-allele (that is, those with an A/C or a C/C genotype) is larger than that of A/A individuals.^7^ A series of studies by Woudstra *et al.* demonstrated that the *PCLO* C-allele increases the vulnerability of an individual to develop MDD by regulating regional brain function that responds to salient stimulation and processes negative information.^8, 9^ Ryan *et al.*^10^ recently reported that *PCLO* rs2522833 was also associated with the volume of the gray matter (GM), and to a lesser extent with hippocampal volume and white matter lesions in patients with late-life MDD. *PCLO* SNP rs2522833 influences the hyperactive response of the HPA system to treatments with antidepressant drugs observed in depressed patients, referred to as inpatients. The *PCLO* SNP rs2522833 C-carriers showed a larger HPA dysregulation than A/A individuals.^11^ They also showed a blunted cortisol awakening response (the rise in cortisol levels during the first hour after awakening).^12^ Previous studies provided evidence for cortisol hypersecretion in patients with MDD.^13^ In addition, HPA axis abnormalities have been observed in these patients, as well as the normalization of HPA axis activity in conjunction with clinical responses to pharmacotherapy.^14^ Thus, HPA axis abnormalities might play a role in the pathophysiology of MDD, which may be why C-allele carriers show an increased risk of developing depression. Excessive cortisol levels are known to have a neurotoxic effect and to reduce the expression of brain-derived neurotrophic factor, a molecule associated with neurogenesis and neuroplasticity in the brain.^15, 16^

According to a meta-analysis, reduced hippocampal volume was reported in subjects with MDD whose duration of illness was longer than 2 years.^17^ Broader networks exist with which the perirhinal cortex, hippocampus and temporal poles interact.^18^ Recently, the ENIGMA Major Depressive Disorder Working Group reported that adult patients with MDD showed a reduced cortical GM volume in the orbitofrontal cortex, anterior and posterior cingulate, insula, and temporal lobes compared to control subjects.^19^ A voxel-based morphometry (VBM) study showed an inverse correlation between cortisol levels and volume reduction in the anterior cingulate cortex in subjects with MDD.^20^ Recently, we also demonstrated that the thickness of the left lateral orbitofrontal cortex was significantly reduced in patients with MDD compared to healthy subjects, and a significant inverse correlation with serum cortisol levels was shown in drug-naive, first-episode patients with MDD.^21^ To the best of our knowledge, no reports have yet investigated the relationship between *PCLO* SNP rs2522833 and morphological changes within the brain in patients with MDD.

In the present study, we used the Statistical Parametric Mapping (SPM) method to investigate the relationships among cortical thinning, rs2522833 SNPs and C-carrier versus A/A genotype in first-episode, drug-naive patients with MDD.

## Materials and methods

The protocol used in this prospective study was approved by the Institutional Review Board of the institution. All participants provided written informed consent to participate in the study. A majority of the subjects in this study participated in an earlier published study;^21^ the main aim of which was to examine the relationship between cortical thickness and serum cortisol levels in MDD using the surface-based morphometry analysis with the FreeSurfer software program (http://surfer.nmr.mgh.harvard.edu).

Thirty right-handed treatment-naive patients experiencing their first episode of MDD were recruited. A psychiatrist (RY) with 28 years of experience in the field used the Structured Clinical Interview according to the Diagnostic and Statistical Manual of Mental Disorders-IV-TR criteria to diagnose a major depressive episode. The severity of depression was evaluated using the 17-item Hamilton Rating Scale for Depression. Only those patients with a Hamilton Rating Scale for Depression total score ⩾14 were eligible for the study. Exclusion criteria included any history of neurological diseases or other physical diseases, and the presence of other disorders (evidence of schizoaffective disorder, bipolar disorder, Axis II personality disorders or mental retardation).

Thirty patients with MDD and 48 healthy controls were further divided into following groups according to *PCLO* genotype: 13 MDD patients with A/A, 17 MDD patients with A/C, 9 healthy controls with A/A, 31 healthy controls with A/C and 8 healthy controls with C/C. We performed this categorization (C-allele, consisting of C/C and A/C, and A/A) because of the previous finding describing differential HPA activity for each group (Table 1).

### Genotyping

Each of the 78 participants provided a blood sample from which the DNA was extracted, and *PCLO* DNA was amplified according to standard laboratory protocols.^22^ In brief, DNA was isolated from peripheral blood mononuclear cells using the QIAamp DNA Mini-Kit (Qiagen, Tokyo, Japan). The human piccolo presynaptic cytomatrix protein (PCLO, Gene ID: 27445) gene was amplified from the genomic DNA using PCR. The PCR products were purified enzymatically. Sequencing reactions were performed using the Big Dye Terminator v3.1 Cycle Sequencing Kit (Life Technologies, Tokyo, Japan). The sequencing primers used were the same as the PCR primers. The sequences were analyzed with the Applied Biosystems 3730xl DNA Analyzer (Life Technologies). The sequencing output data were then compared to a reference sequence (NC_000007.13).

### Plasma cortisol assay

During 30–60 min after awakening, there is a surge of cortisol secretion.^23^ Previous studies showed that patients with MDD exhibit high morning cortisol levels.^24, 25^ Therefore, morning blood samples for a cortisol assay were drawn at 1 h after awakening. Blood samples were drawn from all participants in the morning (0900–1000 hours) and immediately centrifuged (2000 *g*). Plasma samples were stored at −20 °C until they were assayed. The precipitation of proteins with ethanol was followed by a direct radioimmunoassay using a highly specific antibody.^26^

### Neurocognitive tests

Verbal fluency performance in MDD patients was assessed using the Japanese version of the Brief Assessment of Cognition in Schizophrenia. The Brief Assessment of Cognition in Schizophrenia is a reliable and practical scale for evaluating cognitive functions in patients with schizophrenia or mood disorders.^27^ We used total scores from the two trials.

### Magnetic resonance imaging and image processing for VBM

All patients with MDD underwent brain magnetic resonance imaging before receiving antidepressant medication or psychotherapy. Therefore, all participants were antidepressant-free at the time of magnetic resonance imaging. Magnetic resonance imaging data were obtained using a 3.0 T scanner (Signa EXCITE 3T; GE Medical Systems, Milwaukee, WI, USA) with a three-dimensional fast spoiled gradient-recalled acquisition in the steady state. The following parameters were used: repetition time/echo time/inversion time, 10/4.1/700 ms; flip angle, 10º field of view, 24 cm; section thickness, 1.2 mm; and resolution, 0.9 × 0.9 × 1.2 mm. All images were corrected for image distortion due to gradient non-linearity using ‘GradWarp’^28^ and for intensity inhomogeneity using ‘N3’.^29^

Although FreeSurfer software has also been widely used, an equipped surface-based statistical method with this software cannot be used to analyze the subcortical regions. In contrast to FreeSurfer, the standard method of VBM is performed to determine group differences in regional GM volume by measuring tissue composition (GM density). One advantage of SPM compared to FreeSurfer is the possibility to perform statistical analyses in both the whole-cortex and subcortical nuclei, segmented as GM at the same time. Thus, we used volume-based morphometry with SPM. For VBM image processing, SPM8 was used. The three-dimensional fast spoiled gradient-recalled images in native space were spatially normalized to cerebrospinal fluid images and modulated for intensity using DARTEL (Diffeomorphic Anatomical Registration Through Exponential Lie Algebra) toolbox in SPM8.^30^

### Statistical analysis

For the analysis of demographic and clinical characteristics of the participants, an analysis of variance test was performed to test for differences in age, gender and the total GM volume among healthy controls with A/A, healthy control C-carriers, MDD with A/A and MDD C-carriers.

In the VBM analysis, statistical analyses were performed using the SPM8 software. Morphological changes in the GM were assessed using a full factorial model with diagnosis and genotype status (A/A or C-carrier) as independent variables. Age, gender and total GM volume were included as covariates of no interest in all analyses to control for confounding variables. The following *t*-test comparisons were made: (a) effects of diagnosis in A/A individuals; A/A with MDD versus healthy control A/A subjects and (b) effects of diagnosis in C-carriers; C-carriers with MDD versus healthy control C-carriers. All these comparisons were performed on data from the whole brain. These analyses yielded statistical parametric maps {SPM (t)} based on a voxel-level height threshold of *P*<0.001. We used family-wise error correction, setting the significance level at family-wise error-corrected *P*<0.05.

To assess the interaction between the genotype and the effects of diagnosis, we added the analyses of the region of interest containing significant clusters detected using the VBM analysis. In this analysis, we measured brain volume via region of interest and compared the differences between the effects of diagnosis in A/A versus C-carrier individuals by using an analysis of covariance. These statistical analyses were performed using the statistical software package StatView 5.0 (SAS Institute, Cary, NC, USA). A *P*-value of <0.05 was assumed to indicate a statistically significant difference, except in the SPM8 analysis.

## Results

### Demographics and clinical data

The genotypes of 48 control subjects were as follows: 9, *PCLO* rs2522833 (A/A); 31, A/C; and 8, C/C. The A/C-allele frequencies were within the Hardy–Weinberg equilibrium (*X*^2^=4.099, *P*=0.129). The distributions of genotypes A/A, A/C and C/C were 19%, 65% and 17%, respectively.

The genotypes of 30 patients with MDD were as follows: 13, *PCLO* rs2522833 (A/A); 17, A/C; and 0, C/C. The A/C-allele frequencies were within the Hardy–Weinberg equilibrium (*P*=0.468). The distributions of genotypes A/A, A/C and C/C were 43%, 57% and 0%, respectively.

Demographic and clinical information is shown in Table 1. As indicated by one-way analysis of variance (Bonferroni correction), there were no significant differences among the four groups (healthy controls with A/A genotype, healthy control C-carriers, A/A genotype with MDD and C-carriers with MDD) in age (*P*=0.840) nor in GM volume (*P*=0.540).

Moreover, there were no significant differences among the four groups in gender (*P*=0.09), as revealed by the *X*^2^-test, nor in cortisol levels (*P*=0.182), as revealed by the Kruskal–Wallis test.

Plasma cortisol levels were significantly higher in patients with MDD than in the controls (12.07±5.29 nmol l^−1^ versus 9.30±3.43 nmol l^−1^, *P*=0.015). No significant differences were detected in cortisol levels between A/A and C-allele carriers (10.4±3.7 versus 10.5±4.7 nmol l^−1^). Plasma cortisol levels were significantly higher in C-allele carriers with MDD than in C-allele carrier healthy controls (9.31±3.60 versus 12.72±6.11 nmol l^−1^, *P*=0.045).

### The effects of diagnosis

**Figure 1:**
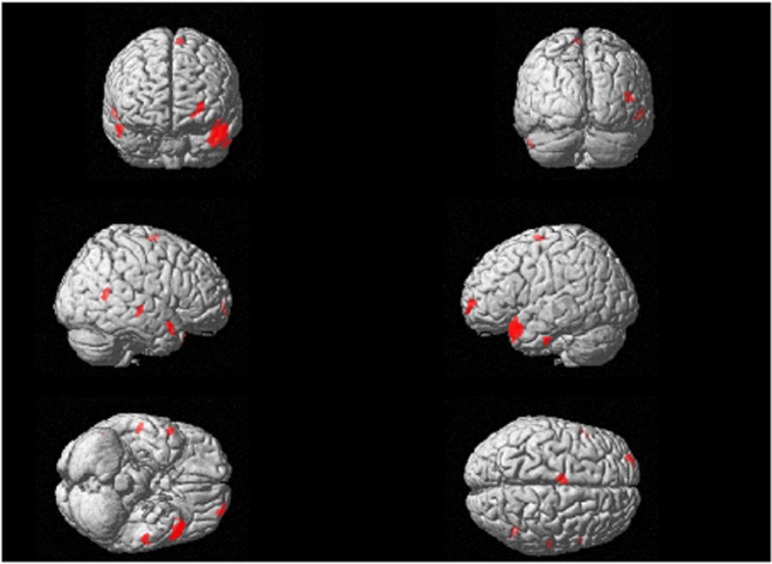
The effects of diagnosis in C-carriers (MDD<healthy controls). The SPM {t} map is displayed in a standard format, as a maximum-intensity projection viewed from the right, the back and the top of the brain. The volume of the left temporal pole was smaller in patients with MDD than in healthy controls (family-wise error-corrected *P*=0.003, *t*=4.96). MDD, major depressive disorder; SPM, Statistical Parametric Mapping.

Among A/A individuals, there were no significant differences in GM volume of any brain region between healthy controls and subjects with MDD. By contrast, among C-carriers, the left temporal pole was significantly smaller in patients with MDD than in healthy controls (family-wise error-corrected *P*=0.02; Table 2; Figure 1). Thus, using region of interest methods, we measured the brain volume of the left temporal pole that detected with the VBM analysis. Regarding volume reduction in the left temporal pole, the genotype–diagnosis interaction analyses demonstrated that the effects of diagnosis were larger in C-carrier individuals than in A/A individuals (*P*=0.0099).

### Volume analysis and plasma cortisol concentrations

**Figure 2:**
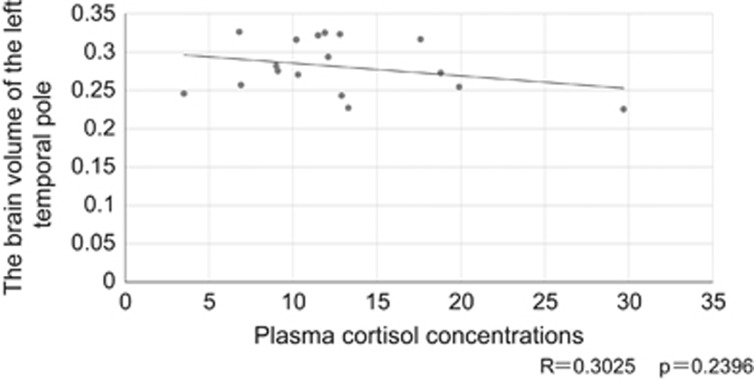
Lack of a strong relationship between the volume of the left temporal pole and plasma cortisol concentration of patients with major depressive disorder carrying the C-allele. *R*=0.3025, *P*=0.2396.

No brain regions showed a correlation between cortical volume and plasma cortisol concentrations in the whole sample, or within the *PCLO* rs2522833 C-carriers and the A/A individuals. Furthermore, no correlation was found between plasma cortisol concentrations and the cortical volume in MDD patients carrying the C-allele (Figure 2).

### Neurocognitive tests

No significant differences were observed between patients with MDD with A/A genotype and those with C-carrier genotype regarding the verbal fluency scores (42.3±8.8 versus 38.3±11.4, *P*=0.328).

## Discussion

To date, this is, we believe, the first study to investigate the association between *PCLO* rs2522833 and GM abnormalities in drug-naive, first-episode patients with MDD. The main finding of the present study was that the volume of the left temporal pole was smaller in C-carriers with MDD than in healthy C-carriers.

The most rostral portion of the temporal cortex, the temporal pole, is known to play an important role in language, multisensory integration, social affective behavior and the theory of mind, the processes associated with mental disorders including mood disorders or schizophrenia.^31^ The results of a recent meta-analysis demonstrated that patients with MDD exhibited a larger cortical volume reduction in the left temporal lobe compared to healthy controls.^19^ Furthermore, electroconvulsive therapy increased the cortical thickness in the bilateral temporal pole in treatment-refractory patients with MDD.^32^ Thus, the temporal pole is considered as one of the regions that is important in MDD. The left temporal pole is considered as the primary site of lesion in semantic dementia, a subtype of frontotemporal lobar degeneration. Thus, we further investigated verbal cognitive functions between C-carrier and A/A subgroups of patients with MDD. No differences were detected between the two groups.

In our study, plasma cortisol levels in patients with MDD were significantly higher than those in healthy controls. Moreover, regarding C-carriers, plasma cortisol levels were significantly higher in the patients with MDD than in healthy controls. Thus, it is plausible that cortisol secretion is enhanced in patients with MDD carrying the C-allele. This is consistent with previous findings that, after the administration of dexamethasone, the HPA axis suppresses the production of CRH by the hypothalamus and adrenocorticotropic-releasing hormone by the pituitary gland, gradually leading to a reduction in cortisol secretion. The lack of suppression of cortisol secretion after a dexamethasone suppression test challenge indicates a dysregulation in HPA axis negative feedback.^12^ Such dysregulation appears to reflect a tonic HPA axis functioning and is usually considered as an indication of hypercortisolemia. By contrast, in the same study, the exogenous infusion of CRH induced a rapid release of adrenocorticotropic-releasing hormone by the pituitary gland, followed by an increase in cortisol production by the adrenal gland. The relative suppression of adrenocorticotropic-releasing hormone and cortisol release after CRH infusion indicates a blunted response of the pituitary gland to CRH. Non-stress-induced basal cortisol levels obtained at key hours of the day may reflect distinct components of the HPA axis. The dysregulation of the HPA axis feedback system, oversensitivity to CRH or adrenocorticotropic-releasing hormone, and/or differences among the patients with MDD could lead to an increase in plasma cortisol levels.

Kuehner *et al.*^12^ demonstrated that *PCLO* rs2522833 was related to a dynamic component of the cortisol awakening response and the blunted cortisol awakening response increase in C-allele carriers. Taken together, their data suggest that C-carriers might have exhausted the inhibitory mechanisms underlying the HPA axis. In addition, the authors of the study demonstrated an association between neuroticism, a premorbid character of MDD or vulnerability to MDD, and the rs2522833 risk variant.^6^ Plasma cortisol levels were higher in patients with MDD than in healthy controls in the present study, particularly in those carrying the C-allele. Taking these findings into account, it appears that patients with MDD carrying the C-allele exhibit a more strongly disturbed HPA axis regulation, which might be associated with the reduction in GM volume of the left temporal pole. The reduction in GM volume of the left temporal lobe in the presence of C-allele was not associated with either the total score of Hamilton Rating Scale for Depression or the categorical sub-score of Hamilton Rating Scale for Depression by Seretti *et al.* (Table 3). This finding suggests that the volume reduction in the GM of this brain region is not associated with the severity of depressive state or any specific symptoms.

Recently, we reported a volume reduction in the thickness of the left lateral orbitofrontal cortex and a significant inverse correlation with serum cortisol levels in patients with MDD.^21^ We did not, however, find a correlation between the volume reduction in the left temporal pole and the plasma cortisol levels in patients with MDD carrying the *PCLO* SNP rs2522833 C-allele. Although glucocorticoid receptor messenger RNA is abundantly distributed in the frontal cortex and the medial temporal lobe,^33^ we found the volume reduction only in the left temporal pole. The precise mechanism underlying the present findings, however, remains unclear. Factors others than the distribution of glucocorticoid receptors might be responsible for the volume reduction in the left temporal pole. Interestingly, Lopes *et al.*^34^ recently reported that dexamethasone significantly reduces the levels of phosphorylated Ca^2+^/calmodulin-dependent protein kinase II and the phosphorylation state of a subtype of α-amino-3-hydroxy-5-methyl-4-isoxazolepropionic acid receptors (GluA1) site at Ser831 in the amygdala of mesial temporal lobe epilepsy related to hippocampal sclerosis patients in a dose-dependent manner. The authors indicated that signal transduction molecules and synaptic neuroplasticity in the limbic system play a role in the effects of glucocorticoids in the human brain under physiological conditions and upon pharmacological manipulation. The prefrontal cortex, amygdala and hippocampus are considered as key brain areas of the feedforward and feedback networks that mediate the states of stress and fear. Glucocorticoids released under stress conditions exert feedforward effects in the whole brain, with a particular importance in limbic structures.^35^ Under stress-free conditions, the prefrontal cortex exerts a top–down regulation of limbic structures including the amygdala, but under acute stress, bottom–up processes prevail, and the behavior changes from slower, highly flexible responses to a faster, stereotyped reaction.^36^ Taking all these findings into account, it appears complicated to pinpoint the exact mechanisms underlying the correlation between the *PCLO* genotype and the left temporal pole volume reduction in C-carriers with MDD. Signal transduction in glutamatergic neurons and/or stress conditions in the patients might have influenced the results in the present study. A further integrative study is needed to precisely elucidate the mechanisms underlying the present result.

This study presents a few limitations. First, it has a relatively small cohort size of patients with MDD, which might have affected the findings. Second, the mechanism underlying the regulation of the HPA axis by risk allele of *PCLO* rs2522833 and the reduction of the left temporal pole volume has not been elucidated. Third, we did not evaluate the cognitive functions in the subjects. A further integrative study taking into consideration the above-mentioned limitations should be performed to confirm the preliminary findings. In addition, the selection of the software for the analyses might have affected our results. Although our previous study using FreeSurfer software demonstrated the correlation between the thickness of the left lateral orbitofrontal cortex and serum cortisol levels in patients with MDD,^21^ in the current study using SPM, we found no correlation between the GM volume and serum cortisol levels. The results of these two studies, therefore, appear inconsistent, which might be explained by differences in registration and statistical approaches between the two software used (FreeSurfer and SPM). The registration method used in Freesurfer is surface-based (vertex-based) rather than volume-based (voxel-based) as in SPM, and the differences in the results arising from these two registration methods have been previously demonstrated.^37^ The association between the *PCLO* rs2522833 genotype, the regulation of HPA axis and the alterations in GM volume and temporal lobe morphology might be an interesting direction for further studies. In conclusion, *PCLO* rs2522833 is associated with a reduction in the volume of the left temporal pole GM.

## Figures and Tables

**Table 1:** Demographic and clinical data

*Diagnoses*	*HS with A/A*	*HS with C-carrier*	*MDD with A/A*	*MDD with C-carrier*	P*-value*
Male/female	4/5	31/8	8/5	9/8	0.090^a^
Age (mean±s.d.)	38.9±12.3	41.6±11.0	47.8±12.4	42.8±12.6	0.840^b^
Corticol levels (mean±s.d.)	9.28±2.84	9.31±3.60	11.22±4.07	12.72±6.11	0.182^c^
Gray matter volume (mean±s.d.)	698.2±77.9	699.0±56.1	682.7±61.6	658.8±63.4	0.540^b^

Abbreviation: MDD, major depressive disorder.

a *χ*^2^-test.

b One-way analysis of variance (Bonferroni correction).

c Kruskal–Wallis test.

Note: mean and s.d.(±) are given.

**Table 2:** Results in SPM8 analysis

*Anatomical regions*	*FWE-corrected* P*-value* *(cluster level)*	*Uncorrected*P *-value* *(cluster level)*	*Cluster size*	T*-value* *(voxel level)*	*Talairach coordinates*		
					x	y	z
Left temporal pole	0.021	0.003	2603	4.96	−46	24	−29
				4.45	−53	16	−25
				3.26	−35	14	−34

Abbreviations: FWE, family-wise error rate; SPM, Statistical Parametric Mapping.

**Table 3:** Left temporal pole volume and HAMD scores of patients with MDD

*Diagnoses*	*MDD with A/A*	*MDD with C-carrier*	P*-value*
Left temporal pole volume	0.32±0.04	0.28±0.04	*P*=0.013
HAMD	20.9±4.5	20.3±6.9	*P*=0.765

*HAMD subscale*			
Core	10.5±3.2	9.2±3.2	*P*=0.310
Sleep	3.2±1.1	3.5±1.3	*P*=0.400
Activty	3.8±1.4	3.8±1.3	*P*=0.871
Psychic	2.6±1.3	2.2±1.6	*P*=0.487
Somatic	3.5±1.2	3.4±1.7	*P*=0.744
Sleep	1.5±1.2	1.6±0.9	*P*=0.863

Abbreviations: HAMD, Hamilton Rating Scale for Depression; MDD, major depressive disorder.

*Note: mean and s.d.(±) are given.
